# The impact of heatwaves on maternal, neonatal and child health outcomes in Western Australian climate zones: A protocol paper for a retrospective cohort study

**DOI:** 10.12688/wellcomeopenres.25022.2

**Published:** 2026-07-25

**Authors:** Erin Kelty, Aster Gebremedhin, Kerrie Mengersen, Shane K Maloney, Peter Le Souef, Michael Kearney, Alexander Larcombe, Amina Rhaman, Melinda Judge, Aiden Price, Kristoffer Wild, Sotiris Vardoulakis, Mara West, Western Australian Healthy Environments And Lives Aboriginal Steering Group (WA HEAL ASG), Caitlin Wyrwoll

**Affiliations:** 1 The University of Western Australia School of Population and Global Health, Perth, Western Australia, 6009, Australia; 2 The University of Western Australia School of Human Sciences, Perth, Western Australia, 6009, Australia; 3 Queensland University of Technology School of Mathematical Sciences, Brisbane, Queensland, 4000, Australia; 4 The University of Western Australia Medical School, Perth, Western Australia, 6009, Australia; 5 Children’s Respiratory Science, The Kids Research Institute Australia, Nedlands, Western Australia, 6009, Australia; 6 The University of Melbourne School of BioSciences, Melbourne, Victoria, 3010, Australia; 7 Curtin University School of Population Health, Perth, Western Australia, 6102, Australia; 8 Wal-yan Respiratory Research Centre, The Kids Research Institute Australia, Nedlands, Western Australia, 6009, Australia; 9 The University of Western Australia Department of Mathematics and Statistics, Perth, Western Australia, 6009, Australia; 10 Australian Urban Research Infrastructure Network, University of Melbourne, Melbourne, Victoria, 3010, Australia; 11 HEAL Global Research Centre, Health Research Institute, University of Canberra, Canberra, Australian Capital Territory, 2610, Australia; 12 The Kids Research Institute Australia, Nedlands, Western Australia, 6009, Australia

**Keywords:** Climate change, environmental health, heat stress, neonatal health, pregnancy.

## Abstract

**Background:**

Rising global temperatures have resulted in an increase in the frequency and severity of heatwaves. Pregnancy is a time of substantial physiological change and thus maternal and neonatal health outcomes are susceptible to adverse effects of heat. There is a lack of knowledge about thermoregulation in pregnancy, including how effectively pregnant people adapt in different climate contexts and what specific environmental conditions present the largest thermoregulatory challenges. The primary aim of this study is to examine the associations between exposure to heatwaves during pregnancy and maternal, neonatal, and child health outcomes in Western Australia climatic regions.

**Methods:**

All neonates born in Western Australia between 1991 and 2023 will be identified from perinatal records (~960,000 neonates). Perinatal records will be linked to weather data from the ERA5 database using Statistical Area 2 geocoding to identify heatwave exposed pregnancies. Commonly used definitions of heatwave exposure will be tested against three neonatal outcomes (pre-term birth, low birth weight, and congenital anomalies). From this, a single definition will be selected to examine maternal, neonatal, and child health outcomes using linked hospital, emergency department, and education assessment data, as well as data from the Western Australian congenital anomalies and death register. The association between heatwaves during pregnancy and health outcomes will be performed using generalised estimating equations, adjusting for covariates including, but not limited to, cigarette smoking, parity, maternal age, socio-economic status, and Aboriginality where appropriate. Stratification by socio-economic status and geographic region will also be carried out.

**Discussion:**

Examining the association between exposure to heatwaves in pregnancy and maternal, neonatal and child outcomes is important given the increasing frequency of heatwaves. The study will highlight which heat definitions confers the highest vulnerability as well as demographic and geographic subgroups who are particularly vulnerable to the effects of heatwaves. It will provide valuable information for future public health planning, including targets for intervention.

## Plain language summary

Our research aims to examine how exposure to heatwaves in pregnancy can impact the health of the mother and her child. It will do this by identifying babies born in Western Australia between 1991 and 2023 and using climate data to determine if they were exposed to a heatwave during pregnancy. Health records will then be used to compare the health of exposed and non-exposed mothers and babies. This information will help us understand how climate change if impacting pregnancy.

## Introduction

As global temperatures increase, the duration and frequency of heatwave events are intensifying across much of the world, ^1^ posing a significant threat to human health. ^2^ While heatwaves can negatively affect all people, some sub-populations are particularly vulnerable, such as pregnant people. Pregnancy is a state of vulnerability to heat exposure as it is a dynamic period of physiological and behavioural adaptation. ^3^ The fetus is entirely dependent on the mother for heat dissipation and temperature stability, which needs to be maintained within a narrow margin. ^4– 6^ Therefore, if the capacity to transfer heat to the external environment is limited, maternal health, placental development and function, and child health outcomes can be compromised.

Exposure to periods of extreme heat, as experienced in heatwaves, during pregnancy has been associated with a wide range of poor maternal, neonatal, and child health outcomes. In neonates, heat exposure has been associated with pre-term birth, stillbirth, and low birth weight, ^7^ as well as an increased risk of congenital anomalies such as congenital heart defects. ^8– 10^ Emerging evidence also suggests that prenatal heat exposure may have longer-term implications for child health, including increased risk of respiratory conditions such as asthma and pneumonia, as well as cardiometabolic outcomes including elevated blood pressure. ^11^ However, the evidence base for these outcomes remains comparatively limited. For mothers, heat exposure during pregnancy has been associated with an increased risk of gestational diabetes, ^12^ pre-eclampsia ^13^ and hospitalisation during pregnancy. ^14^ The biological drivers that underly the adverse consequences of heat on pregnancy are thought to be multifactorial and outcome dependent. ^15, 16^ However, to date there is little conclusive evidence of definitive mechanisms, although a range of physiological and endocrine changes have been implicated. ^17^ There is also a lack of knowledge on thermoregulation in pregnancy, including how effectively pregnant people adapt in different climate contexts and what specific environmental conditions present the biggest thermoregulatory challenge. In addition to the direct pathways whereby heat can impact pregnancy, there are a number of indirect pathways, including air pollution, infectious diseases, and social or healthcare disruption.

While heatwaves are likely to have a negative effect on maternal and neonatal health outcomes in general, the quantitative effect is likely to vary with socio-demographics, other environmental factors (e.g. humidity, wind speed, and air pollution), and timing/trimester of exposure. In both pregnancy and non-pregnant persons, multiple socio-economic factors have been identified that either exacerbate or mitigate the association between heat exposure and health outcomes. Poverty and low socio-economic status have been associated with an increased risk of mortality during heatwaves in non-pregnant patients, ^18^ likely related to reduced access to air-conditioning and green spaces, both of which can help to mitigate the effects of heat. ^19– 21^ Additionally, socio-economic disparities can shape vulnerability to heat exposure during pregnancy, with associations between heat and preterm birth reported to be strongest among lower socio-economic groups ^22^ among socially marginalised populations, including African American mothers in the United States. ^23^ Similarly, high humidity dramatically increases the physiological strain that is induced by exposure to high temperature, reducing the efficacy of evaporative cooling, while air pollution (including from wildfires) has also been demonstrated to increase the impact of heatwaves on health outcomes. ^24^ The timing of exposure to heat stress has a substantial impact on maternal and neonatal health outcomes, although the impact of timing is highly dependent on the outcome of interest. For example, exposure to a heatwave during the first trimester when organogenesis is occurring has been associated with an increased risk of congenital anomalies, ^8^ while exposure late in pregnancy is unlikely to increase the risk of congenital anomalies. Conversely, third trimester exposure to heat has been associated with an increase in uterine contractility ^25^ and pre-term birth. ^7^

As yet, there is no consensus on what constitutes a heatwave. While the premise is relatively simple, in a 2016 systematic review examining the impact of heatwaves on mortality, 15 different definitions utilising maximum temperature, 13 different definitions using mean temperature, and 16 specific heat events were used in the 60 papers examined. ^26^ Definitions typically vary based on their use of temperature indicators (e.g. maximum daily temperature, mean daily temperature, apparent temperature, and heat index), temperature threshold (e.g. relative threshold or absolute threshold), and duration. Additionally, complex measures can factor in other environmental factors that impact the heat balance in humans, such as humidity and wind speed. The use of different heatwave definitions creates a problem for interpretation, comparability, and translation into practice. While there are likely strong correlations between heatwave definitions, the selection of definitions can impact the strength of association between heat and the outcome of interest. ^26^ Thus, the selection of a definition can be an extremely important consideration, particularly in the context of the study setting. For example, an absolute threshold of 35°C may be appropriate for a single city but not for larger areas that have substantial climate variability or where temperatures are regularly above this threshold. Western Australia provides a unique context to examine heat definitions, as it includes a range of climatic zones (tropical, sub-tropical, dessert, temperate, grassland), ensuring definitions equivalently capture heat exposure in different climates.

The proposed research will examine the relationship between heatwaves and pregnancy, maternal, neonatal, and child health outcomes in Western Australia (WA) between 1991 and 2023. As part of the work, we will examine which heatwave indicators are mostly strongly associated with pregnancy and neonatal outcomes and whether these measures are interrelated. Additionally, we aim to examine how the relationship is affected by climate zone, socio-economic status, environmental factors, and trimester of exposure.

## Protocol

### Setting

Heatwaves are one of the most common natural hazards in WA, and are responsible for more deaths than any other natural hazard including bushfires, floods, storms, and tropical cyclones. ^27^ WA provides a unique setting to examine heatwaves, due to its diversity in climates, ranging from temperate to equatorial ( Figure 1).

**Figure 1.**
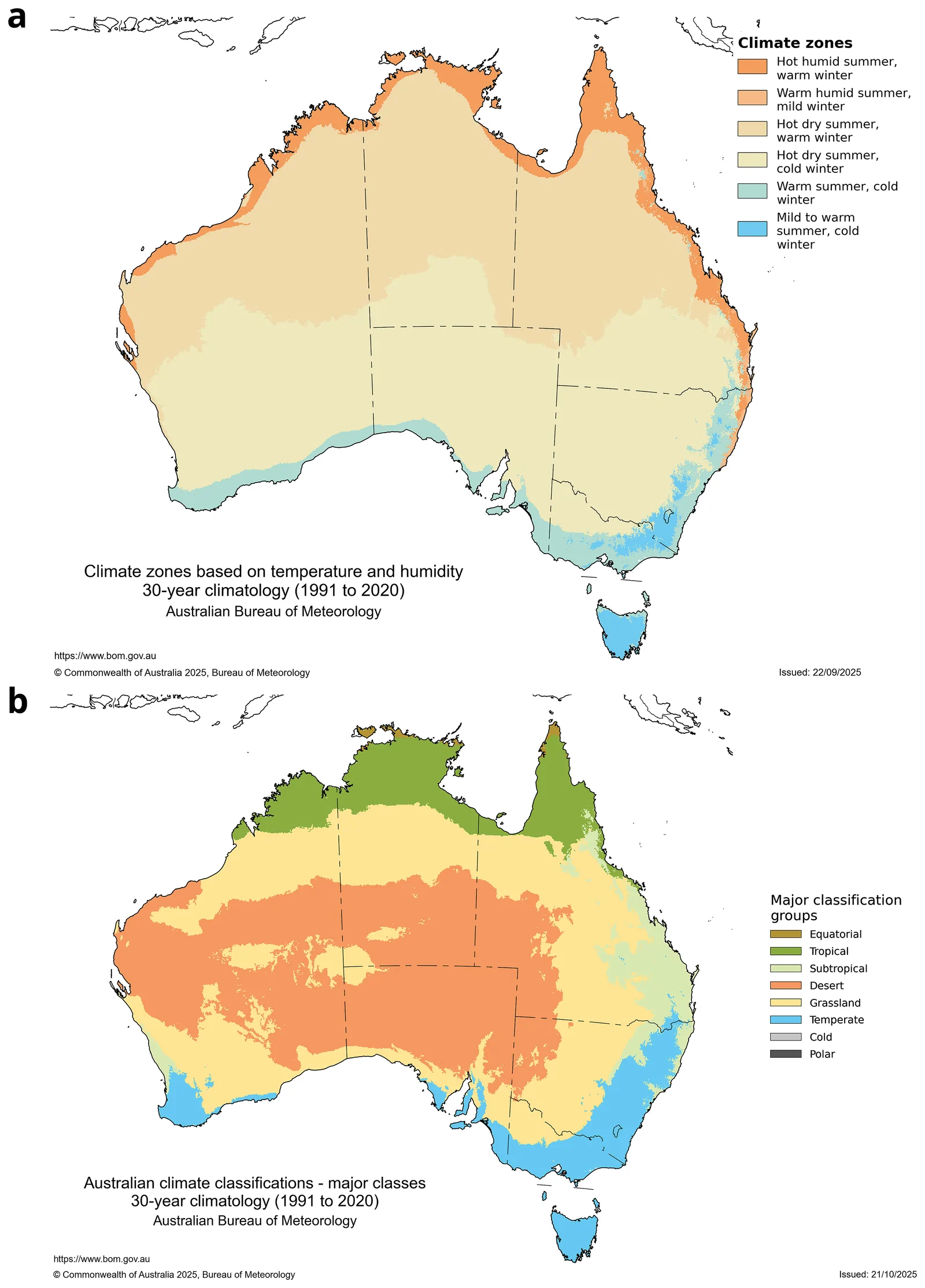
Australian climate zones based on temperature and humidity (top) and the Köppen classification system (bottom). ^28^ Western Australia (state on the far left) comprises of multiple climate zones.

### Population

The study will include all neonates born in WA between 1991 and 2023. This includes all live and stillborn neonates of at least 20 weeks gestation or ≥ 400 grams where gestational age was unknown. We expect that the dataset will include approximately 960,000 neonates.

### Data sources

The study cohort will be identified from state perinatal records (Midwives Notification System), while exposure data will be obtained from ERA5. For this study, outcomes data will be obtained from the PeopleWA. PeopleWA is a newly established linked data asset containing whole population data for the state of WA. It contains data from the WA Departments of Communities, Education, Health, Justice, and the Australian Police Force. For this study, we will primarily utilise data from the Department of Health, including the Midwives Notification System, Hospital Morbidity Data Collection, the Emergency Department Data Collection, and the WA Register of Developmental Anomalies (WARDA). Death data from the Register of Births, Deaths and Marriages (within the Department of Justice) and standardised educational testing data from the Department of Education will also be included.

### Exposure

Climate data from ERA5 will be mapped to birth records using geocoding at the level of Statistical Area 2 (SA2). SA2 are medium sized geographic areas regions consisting of around 3,000 to 25,000 people (average 10,000 people) defined by the Australian Bureau of Statistics. They typically represent a community that interacts together socially and economically. Weather data obtained from the ERA5 database will contain hourly measures of dry-bulb temperature, rainfall, humidity, wind speed, and solar radiation from January 1990 to December 2023, providing climate data for at least one year prior to the birth for all neonates in the health dataset. ERA5 contains historical data at hourly resolution from 1940 to the present in a gridded dataset at 0.25° × 0.25° resolution across all of the land surfaces on earth. ^29^ Climate data will be extracted from the midpoint of each SA2 region within each SA2. For small SA2, located in urban areas near and surrounding Perth, it is expected that the midpoints will provide a good estimate of heat exposure for the SA2. However, in rural and remote areas with much larger SA2 (landmass greater than 10,000 km ^2^) the midpoint is likely to be less representative of the whole SA2. For these SA2, data will also be extracted for the largest town with the SA2 to hopefully better reflect exposure.

Six indices of heat will be used to define a range of heatwave exposure definitions ( Table 1). These indices will range in complexity from dry-bulb temperature which is based solely on air temperature to more complex indices that incorporate other environmental factors that impact on human thermoregulation, up to complete rational heat balance models of human thermoregulation. Each of the indices will be calculated to estimate exposure to heat stress during pregnancy ( Table 1).

**Table 1.** Summary of heatwave indices that will be used to define heatwaves examine the association between prenatal exposure to heatwaves and neonatal outcomes.

No	Heatwave definition/index	Climate variables considered in the index	Advantages and uses	Limitations	Examples of use
1	Ambient temperature	Actual air (dry-bulb) temperature	Easy and commonly used measure	Provides a baseline measure of temperature only (it does not consider other environmental factors)	30
2	Apparent temperature (AT)	Temperature and relative humidity (may also incorporate wind speed)	Common metrics used as indicators of the heat-related stress on the human body (accounts for the combined impacts of humidity and temperature)	Assumes that the population that is exposed to the temperature will be in the shade	31
3	Wet bulb temperature (T _wb_)	Temperature and relative humidity	Used to assess the risk of humid heat and capture the critical role of sweat evaporation	Difficult to interpret and communicate because it combines temperature and humidity in a complex, non-linear way	32
4	Wet Bulb Globe Temperature (WBGT)	Temperature, humidity, wind speed, and solar radiation	Considered as an appealing measure for estimating thermal conditions experienced by outdoor workers and athletes	Underestimation of thermal stress in conditions where sweating is restricted, susceptibility to measurement errors, and variability based on clothing and activity	33
5	Excess Heat Factor (EHF)	Maximum temperature and minimum temperature	Used in Australian heatwave monitoring and forecasting, accounts for acclimatisation and heatwave intensity	Based only on ambient temperature	34
6	Universal Thermal Climate Index (UTCI)	Temperature, humidity, wind speed, and solar radiation (Computed using complex physiological model)	Derived from an advanced human thermoregulation model and incorporates all key thermal factors. High suitability and simulation of the climatic sensitivity of the human body	May not reflect the physiological conditions of pregnant person, as it is based on a standard model that does not account for pregnancy-specific characteristics.	35

Initially, heatwave definitions based on air temperature (also known as ambient or dry-bulb temperature) will be used. These will include:

• Absolute cutoffs: Daily maximum temperatures (T _max_) above 32°C, 35°C, 37°C for 2, 3 and 4 consecutive days.
• Mean temperature: Mean daily temperate (T _mean_) above the 90 ^th^, 95 ^th^ and 99 ^th^ percentile for 2, 3 and 4 consecutive days.
• Maximum temperature: T _max_ above the 90 ^th^, 95 ^th^ and 99 ^th^ percentile for 2, 3 and 4 consecutive days.
• Minimum temperature: Minimum daily temperature (T _min_) above the 90 ^th^, 95 ^th^ and 99 ^th^ percentile for 2, 3 and 4 consecutive days.
• Mean temperature for warm months: T _mean_ for November to March above the 90 ^th^, 95 ^th^ and 99 ^th^ percentile for 2, 3 and 4 consecutive days.
• Maximum temperature for warm months: T _mean_ for November to March above the 90 ^th^, 95 ^th^ and 99 ^th^ percentile for 2, 3 and 4 consecutive days.
• Minimum temperature for warm months: T _min_ for November to March above the 90 ^th^, 95 ^th^ and 99 ^th^ percentile for 2, 3 and 4 consecutive days.
• Nocturnal mean: Daily mean temperature for 20:00–07:00 above the 90 ^th^, 95 ^th^ and 99 ^th^ percentile for 2, 3 and 4 consecutive days.
• Nocturnal mean for warm months: Daily mean temperature for 20:00–07:00 during November to March above the 90 ^th^, 95 ^th^ and 99 ^th^ percentile for 2, 3 and 4 consecutive days.

Nocturnal temperature will also be considered to capture heat exposure during nighttime hours. Elevated temperatures at night, particularly minimum temperatures, can impair overnight physiological recovery and may be especially relevant in pregnancy. ^36, 37^

These temperature-based definitions, which are widely used in the literature, ^30, 38, 39^ will serve as a reference for more complex models that incorporate additional climate parameters such as humidity, solar radiation, wind speed, and other variables that capture heat exposure more comprehensively.

Subsequently, definitions based on apparent temperature (AT) and wet bulb temperature (T _wb_) will be applied to account for the combined effects of temperature and humidity. The apparent temperature is a composite biometeorological index that is calculated using air and dew-point temperature. The dew-point temperature is the temperature at which water will condense from the air at the prevailing humidity. The apparent temperature will be calculated using the following two formulas.

1)
$AT=2.653+(0.994\times T_{a})+0.0153\times Td^{2}),$ where *T _a_* is the ambient temperature (°C) and *T _d_* is the dew-point temperature (°C) ^40^
2)
$AT=T_{a}+0.33\times e-0.70\times Ws-4.00$ where $e=\frac{Rh}{100}\times 6.11\times \exp(17.27\times \frac{T_{a}}{\left(237.7+T_{a}\right)})$ and *T*_*a*_ is the ambient temperature (°C); *e* is actual vapor pressure (hPa); *Rh* is relative humidity (%); and *Ws* is wind speed (m/s). ^41^

The Australian Bureau of Meteorology uses formula 2.

Wet bulb temperature has been identified as a relevant metric to assess heat-related health risks, as it captures the critical role of sweat evaporation. Applying the Stull method formula, it is calculated as follows. ^32, 42^

Twb=Tdb×atan{0.151977×(RH+8.313658)12}+atan(Tdb+RH)−atan(RH−1.676331)+0.00391838×RH32×atan(0.023101×RH)−4.686035

Where relative humidity (RH) is defined as.

$RH=0.263\times PRES\times SH\times {\left\{\exp(\frac{17.67\times (T_{db}-0.01)}{T_{db}-243.5})\right\}}^{-1},\;T_{wb}$ and *T _db_* denotes the wet-bulb and dry-bulb temperatures, respectively, and *PRES* denotes air pressure.

Wet-bulb temperature ( *T _wb_*) can be estimated from electronic measures of dry-bulb temperature ( *T _db_*) and relative humidity ( *RH*). However, the formulae available for deriving *T _wb_* from *T _db_* and *RH* provide only approximations and may be inaccurate under certain conditions. While RH can be easily derived from *T _db_* and *T _wb_*, the reverse calculation from *T _db_* and *RH* to *T _wb_* is not algebraically possible and therefore requires an iterative process. In the iterative approach, the following formulae from Barenbrug (1974) ^43^ are applied.

The saturation water vapour pressure in kPa ( *e _sat_*) is calculated as

$$e_{sat}=0.6106*exp\left[\left(\frac{17.27*T_{db}}{237.3+T_{db}}\right)\right]$$ 1)

Where *exp* is the base of natural logarithms ( *e*) to the power of the number in the square brackets.

The water vapour pressure in an unsaturated atmosphere in kPa ( *e*) is therefore (where *RH* is expressed as a proportion)

$$e=e_{sat}*RH$$ 2)

The relationship between *e* and the *T _db_* and *T _wb_* at any given atmospheric pressure ( *BP*) is given by

3) $$e=\frac{\left[e_{sat*}(371.4+0.24*T_{db}-0.6*T_{wb})-0.24*(T_{db}-T_{wb})*BP\right]}{371.4+0.04*T_{db}-0.4*T_{wb}}$$

In the iterative process, *e* is obtained using formulae 1 and 2, and then formula 3 is solved iteratively to obtain *T _wb_*. Standard barometric pressure ( *BP*) is assumed as 101.3 kPa.

Another definition will be based on Wet Bulb Globe Temperature ( *WBGT*), a composite index of thermal stress that better captures human physiological responses to outdoor heat. It is determined from readings of the natural wet-bulb temperature ( *T _nwb_*), black globe temperature ( *T _g_*), and air temperature ( *T _a_*), weighted as follows ^44, 45^:

For outdoor environments, $WBGT=0.7T_{nwb}+0.2T_{g}+0.1T_{a}$ .

For indoor environments (where there is no direct solar radiation and the globe temperature is usually similar to the dry-bulb temperature), $WBGT=0.7\times T_{nwb}+0.3\times T_{g}$ .

For both AT and *WBGT*, heatwaves will be defined using *T _mean_* and *T _max_* for the whole year and warm months using cutoffs of 90 ^th^, 95 ^th^ and 99 ^th^ percentile for 2, 3 and 4 consecutive days. For AT, absolute cutoffs of 32°C, 35°C, 37°C will also be used.

The Excess Heat Factor ( *EHF*), which underpins the Australian Bureau of Meteorology’s national heatwave forecasting service, will be applied. *EHF* combines excess heat, which reflects long-term temperature anomalies relative to the local climatological distribution, and heat stress, which captures short-term anomalies based on recent physiological thermal acclimatisation. It provides a relative measure of the load, intensity, spatial extent, and duration of a heatwave event. As shown below, the *EHF* calculation compares the daily average temperature ( *DAT*) for 3 days with the historical temperature (95th percentile for historical 30-year temperature data (DMT _95_)) for that area, known as the significant excess heat index ( *EHI _sig_*), and the *DAT* for the preceding 30 days known as heat stress or acclimatisation excess heat index ( *EHI _accl_*). ^46^

$$EHF=EHI_{sig}\times \max(1,\;EHI_{accl}),$$

Where $DAT=\frac{\left(DAT_{max}+DAT_{MIN}\right)}{2},$ and

$$EHI_{sig}=\frac{\left(DAT_{0}+DAT_{1}+DAT_{2}\right)}{3}-DAT_{95th}$$

$$EHI_{accl}=\frac{\left(DAT_{0}+DAT_{1}+DAT_{2}\right)}{3}-\frac{\left(DAT_{-1}+\cdots +DAT_{-30}\right)}{30}$$

According to the Bureau of Meteorology (BOM), a day is classified as a heatwave day if the Excess Heat Factor ( *EHF*) exceeds zero; otherwise, it is considered a non-heatwave day. ^34^ Other studies have categorised heatwaves into different intensity levels: low intensity ( *EHF* > 0 and < 1), moderate intensity ( *EHF* ≥ 1 and < 2), and high intensity ( *EHF* > 2). ^47, 48^

We will calculate the Universal Climate Thermal Index (UTCI), an isothermal equivalent air temperature (°C) of the reference condition causing the same human physiological response to the actual thermal environmental condition. It is computed through the integration of key meteorological variables, including air temperature at 2 metres, wind speed at 10 metres above ground level, relative humidity, and the mean radiant temperature. ^49^ The UTCI is classified into ten categories of thermal stress based on defined temperature ranges, each reflecting a distinct level of physiological response to the thermal environment. For heat-related conditions, the categories include moderate heat stress (UTCI from 26°C to 32°C), strong heat stress (32°C to 38°C), very strong heat stress (38°C to 46°C), and extreme heat stress (above 46°C). ^50^ Heatwaves will be defined as maximum daily UTCIs above 26°C, 32°C and 38°C for 2, 3 and 4 consecutive days. Additionally, maximum daily UTCI above the 90 ^th^, 95 ^th^ and 99 ^th^ percentile for 2, 3 and 4 consecutive days will be used to define heatwaves.

### Outcomes

Given the broad nature of the linked data, there is the ability to explore in detail the maternal, neonatal, and child health associated with exposure to heatwaves in pregnancy. To better understand the impact of different heatwave indicators, an exploratory analysis of three neonatal outcomes have been selected: pre-term birth, low birth weight (<2,500 g), and congenital anomalies. These outcomes are the most commonly studied neonatal outcomes, with substantial robust evidence supporting their association with heatwave exposure. ^9^

In examining the association between the singular heat exposure definition and maternal, neonatal and child health a number of primary and secondar outcomes have been selected. Maternal health will be examined using a number of primary and secondary outcomes, with a focus on outcomes that have been associated, or are likely to be associated, with hyperthermia. These will include threatened pregnancy loss, gestational diabetes, pre-eclampsia, the onset of labour (spontaneous, induced, or no labour), and type of delivery (vaginal or caesarean).

The primary outcomes that will be used to examine neonatal health will be perinatal mortality, birth size (birth weight, birth length, length of gestation, and head circumference), and congenital anomalies. The primary outcomes will be supported by secondary outcomes that include percentage of pre-term birth (<37 weeks), very pre-term birth (<32 weeks), extremely pre-term birth (<28 weeks), low birth weight (<2,500 g), very low birth weight (<1,500 g), small for gestational age (<10 ^th^ percentile), admission to the special care unit, and congenital anomalies categorised by the affected system (e.g. cardiovascular, urogenital). Child health outcomes will have a focus on the effects of gestational heat exposure on mental health outcomes, educational scores (literacy and numeracy test scores) and the immune-related conditions (hospital and ED presentations for infection, allergies, and asthma).

When examining outcomes by trimester, outcomes have been selected for examination with perceived biological plausibility. For the first trimester, threatened pregnancy loss and congenital anomalies will be examined, while for the second trimester gestational age, very pre-term birth (<32 weeks), birth weight and admission to the special care unit will be examined. For the third trimester, stillbirth, resuscitation, gestational age, spontaneous and iatrogenic pre-term birth, birth weight, onset of labour, delivery type and admission to the special care unit will be examined.

### Statistical analysis

Each of the selected binary heatwave indicators will be used independently to identify pregnancies that involved exposure to heatwave. Generalised estimating equations will be used to look at the association between each indicator and the three outcomes of interest (pre-term birth, low birth weight, and congenital anomalies), accounting for the mother having multiple neonates within the study. The odds ratio and 95% confidence intervals will be presented for each. Cohen’s Kappa will be used to calculate the level of agreement between different heatwave indicators. From that comparison the research group will select a single heatwave indicator that will be used for subsequent analysis. The selection of this definition will be based on a number of factors, including the strength of association with the predefined outcomes, correlation with other measures, portion of exposed pregnancy identified (overall and by climate zone), ease of interpretation and use within the literature and policy.

Initial comparisons between the exposed and non-exposed group will be made in terms of demographics, including maternal age, smoking status during pregnancy, Aboriginality, and rurality. To examine the association between exposure to heat stress as defined by each of the various indices and health outcomes, generalised estimating equations (GGE) will be used, adjusting for relevant covariates. The use of GEE will allow for multiple births per mother to be clustered in the analysis. The analysis will also be performed stratified by several important variables. These include variables relating to region (e.g. climate and rurality), environmental factors (e.g. seasonality), and patient characteristics (e.g. socio-economic status and year of birth). Characterisation of Aboriginal health outcomes is guided by WA Healthy Environments And Lives (HEAL) Aboriginal Steering Group.

The association between heat exposure and health outcomes will be further examined based on the timing/trimester of exposure. Within these analyses, the outcomes of interest have been limited to outcomes that could feasibly be associated with heat exposure based on the trimester.

## Conclusion

This retrospective cohort study will examine the impact of exposure to heat on pregnancy outcomes over four decades across the diverse climate of Western Australia. The analysis will capture more than 960,000 births, so it is anticipated it will provide sufficient power to examine rare outcomes (e.g. stillbirth) and provide insight into the likely complex and multifaceted relationship between heat stress and pregnancy outcomes, factoring in adaptation to climate zone, socioeconomics, climate, rurality, and timing of exposure.

In carrying out this research, we felt that it was important to first understand how various definitions of heatwaves that have been employed in pregnancy cohort studies differ from each other and their association with common neonatal outcomes. To produce robust and replicable research, it is important that there is a good understanding of how the different measures can impact outcomes and what exposure measures should be preferentially used or not used. It is hoped that moving forward, this research will be used to encourage consistency in the definitions of heatwaves used in examining pregnancy exposure.

The findings from this study will have implications for public health policies and guidelines that relate to heat stress and pregnancy, nationally and globally. These findings will progress a prediction tool of future health burden for each climate zone. By identifying the potential risks that are associated with exposure to heat during pregnancy, more targeted strategies can be developed to mitigate these risks and protect the health of pregnant people and their children. Health impacts from heat in pregnancy are largely preventable but there are fundamental gaps in our understanding of who is most at-risk. Ultimately, this research may help to inform the development of public health policies that are aimed at reducing the negative impact of heat exposure on pregnancy outcomes.

## Ethics

The study has been approved by Department of Health WA Human Research Ethics Committee (RGS0000006684), PeopleWA and each of the data custodians. This project was developed with the support of the WA Healthy Environments And Lives (HEAL) Aboriginal Steering Group. The study was also approved by the WA Aboriginal Health Ethics Committee (HREC1326). A waiver of consent was approved for the project, as the study meets the criteria set out in the National Statement on Ethical Conduct in Human Research. ^51^ Briefly, this required the proposed research to be low risk to participants, with sufficient protection to their privacy. Additionally, it would be impractical to obtain consent from all participants and there was no known reason they would not have consented if asked. The benefits of the research also justified the risk of harm associated with not seeking consent.

## Data availability statement

Individual level data will not be made publicly available due to the potentially sensitive and identifiable nature of the data. This is a condition of both ethical and data custodian approval of the study. Access to participant data can be applied for through PeopleWA ( peoplewa@dpc.wa.gov.au). Environmental data may be provided upon reasonable request.

## Acknowledgements

We acknowledge the critical role of the Western Australian Healthy Environments And Lives Aboriginal Steering Group (WA HEAL ASG) in guiding EHPC; Aunty Mara West, Uncle Noel Nannup, Uncle Roger Turvey, Aunty Virginia Westwood, Lynette Dimer, Jason Barrow, Tia Whyman, Jo Taylor, Jacob West, Liam Bedford, Jacinta Walton and Catherine Holland.
