# Supplementary material for: The impact of heatwaves on maternal, neonatal and child health outcomes in Western Australian climate zones: A protocol paper for a retrospective cohort study
Source: Wellcome Open Res. 2026 Oct 13;11:7. Originally published 2026 Jan 5. [Version 3] doi: 10.12688/wellcomeopenres.25022.3 (PMC13687742; doi:10.12688/wellcomeopenres.25022.3)
Supplement: Peer review history

# Reviewer report: Reviewer response for version 3

**DOI:** 10.21956/wellcomeopenres.30863.r173818

**Published:** 2026-10-28

**Authors:** Cara Schulte

**Affiliations:** 1 University of California Berkeley (Ringgold ID: 1438), Berkeley, California, USA

I am confident the authors have adequately addressed my comments, and I look forward to reading the results of this study.

Is the study design appropriate for the research question?

Partly

Is the rationale for, and objectives of, the study clearly described?

Yes

Are sufficient details of the methods provided to allow replication by others?

Partly

Are the datasets clearly presented in a useable and accessible format?

Not applicable

Reviewer Expertise:

Extreme heat and human health; perinatal and reproductive health; occupational and environmental health; climate adaptation; health and human rights

I confirm that I have read this submission and believe that I have an appropriate level of expertise to confirm that it is of an acceptable scientific standard.

# Reviewer report: Reviewer response for version 3

**DOI:** 10.21956/wellcomeopenres.30863.r173815

**Published:** 2026-10-21

**Authors:** Caroline Li-Maloney

**Affiliations:** 1 Human and Environmental Physiology Research Unit, School of Human Kinetics,, University of Ottawa, Ottawa, Ontario, Canada

I am satisfied that the authors have taken the time to address previous concerns I had about the methodology and have clarified the use of heatwave definitions (including those that address humidity) and exposure windows. Although I had concerns about the inclusion of pediatric outcomes, I appreciate that the authors have provided better context for this decision. I feel that this project has become quite ambitious given the number of outcomes and metrics employed, however, if the authors are able to apply this level of rigor I do feel it will be a valuable contribution to the field.

Is the study design appropriate for the research question?

Partly

Is the rationale for, and objectives of, the study clearly described?

Yes

Are sufficient details of the methods provided to allow replication by others?

Yes

Are the datasets clearly presented in a useable and accessible format?

Not applicable

Reviewer Expertise:

Thermal physiology specializing in female health outcomes, with a focus on pregnancy and post-menopause.

I confirm that I have read this submission and believe that I have an appropriate level of expertise to confirm that it is of an acceptable scientific standard.

# Reviewer report: Reviewer response for version 2

**DOI:** 10.21956/wellcomeopenres.30075.r163073

**Published:** 2026-08-26

**Authors:** Cara Schulte

**Affiliations:** 1 University of California Berkeley (Ringgold ID: 1438), Berkeley, California, USA

*The protocol proposes a retrospective cohort study in which weather data from the ERA5 database will be linked to perinatal records in Western Australia from between 1991 and 2023. The first objective is to test a number of varying heatwave definitions against three birth outcomes: PTB, LBW, CA.*

*Using this data, researchers will select a single heatwave definition to later apply to a wider range of outcomes. In the second phase of the study, researchers will use generalized estimating equations to apply the chosen definition to a range of maternal, neonatal, and child health outcomes. Findings will be stratified by economic status and geographic region.*

*The study will address a key question in the existing literature in comparing multiple heat indices and heatwave definitions. In addition to examining fetal health outcomes, the study will also include maternal health outcomes, which are generally underrepresented in the heat-health literature.*

*Please find my questions and suggestions below:*

***COMMENTS ON METHODOLOGY***

*- One of my main concerns is that conducting multiple analyses on the same data set (with many differing heatwave definitions) will increase the probability of finding statistically significant results. Please explain how your methodology intends to reduce the risk of false positives.*

*- “Climate data will be extracted from the midpoint of each SA2 region within each SA2. For small SA2, located in urban areas near and surrounding Perth, it is expected that the midpoints will provide a good estimate of heat exposure for the SA2. However, in rural and remote areas with much larger SA2 (landmass greater than 10,000 km2) the midpoint is likely to be less representative of the whole SA2. For these SA2, data will also be extracted for the largest town with the SA2 to hopefully better reflect exposure.” Proxy temperature data is often used in the heat-health literature, despite known challenges. Within urban areas with microclimates, temperature data becomes less accurate within a few kilometers. In more rural areas, it is less accurate after about 25 km. Is it possible to use a more accurate proxy measure than midpoint in each of these regions? If not, please include discussion of this decision (including data availability challenges) in your limitations.*

*- Because the study will not account for indoor temperatures, occupational exposure, or climate adaptation measures (such as residential air conditioning, which is common in over 50% of homes in Western Australia), please also include discussion of this as a limitation.*

*- Please indicate which exposure windows will be used for measuring maternal health outcomes and the physiological grounding for such decisions.*

*- “This includes all live and stillborn neonates of at least 20 weeks gestation or ≥ 400 grams where gestational age was unknown.” Please add discussion of how you intend to link temperature/heat stress data with fetuses of unknown gestational age, if at all.*

*- If any of the birth record data is self reported, or where gestational age is measured via last menstrual period (LMP) vs. ultrasound, please indicate how you will address any potential biases that may arise.*

*- If the chosen heat index falls within category 1 (ambient temperature) or 2 (excess heat factor), how will you justify dropping humidity from your analysis? I am particularly concerned about this given that in conditions where relative humidity is > 50% (common in some regions of Western Australia), not accounting for humidity underestimates heat stress. Because we already know that ambient temperature is a crude proxy that can create substantial exposure misclassification, what will you do if this ends up being the selected metric? I would question how that could potentially discount the value of this research.*

*- “The selection of this definition will be based on a number of factors, including the strength of association with the predefined outcomes, correlation with other measures, portion of exposed pregnancy identified (overall and by climate zone), ease of interpretation and use within the literature and policy.” I would suggest outlining clearer criteria for the eventual selection process prior to conducting the initial analysis. As written, it's not entirely clear what you mean by each of these categories or how you intend to weight them in your selection process.*

***LINE EDITS***

*- This is picky, but I would rephrase “There is a lack of knowledge about thermoregulation in pregnancy” to something like “There is incomplete knowledge about” because there is a lot we do know, but of course there are clear gaps which you go on to highlight in the second clause.*

*- “The study will highlight which heat definitions confers the highest vulnerability” - plural subject does not match singular verb.*

*- “In neonates, heat exposure has been associated with pre-term birth, stillbirth, and low birth weight,” - stillbirth is not a neonatal health outcome, nor are stillborns considered “neonates.”*

*- “Additionally, complex measures can factor in other environmental factors that impact the heat balance in humans, such as humidity and wind speed” - might make sense to introduce more technical terms here, such as “Additionally, more complex measures of heat strain can factor in other environmental factors that can alter the net heat load placed on the human body, such as …”*

*- “For these SA2, data will also be extracted for the largest town with the SA2 to hopefully better reflect exposure” - think this should be “within.”*

***RELEVANT PAPERS***

*While I’m sure you’ve seen these, I’m sharing two publications that I did not see cited in the protocol that are likely relevant to your study design:*

*- (Heil C et al.,2026) (new)*

*- (Brimicombe C et al.,2024)*

Is the study design appropriate for the research question?

Partly

Is the rationale for, and objectives of, the study clearly described?

Yes

Are sufficient details of the methods provided to allow replication by others?

Partly

Are the datasets clearly presented in a useable and accessible format?

Not applicable

Reviewer Expertise:

Extreme heat and human health; perinatal and reproductive health; occupational and environmental health; climate adaptation; health and human rights

I confirm that I have read this submission and believe that I have an appropriate level of expertise to confirm that it is of an acceptable scientific standard, however I have significant reservations, as outlined above.

## References

[1] : A systematic review and meta-analysis on heat thresholds for maternal health and birth outcomes. *Nature Communications* .2026;17(1) : 10.1038/s41467-026-76822-8 10.1038/s41467-026-76822-8 PMC1349393942624863

[2] : A scoping review on heat indices used to measure the effects of heat on maternal and perinatal health. *BMJ Public Health* .2024;2(1) : 10.1136/bmjph-2023-000308 10.1136/bmjph-2023-000308 PMC1181275740018117

# Reviewer report: Reviewer response for version 2

**DOI:** 10.21956/wellcomeopenres.30075.r163072

**Published:** 2026-08-06

**Authors:** Caroline Li-Maloney

**Affiliations:** 1 Human and Environmental Physiology Research Unit, School of Human Kinetics,, University of Ottawa, Ottawa, Ontario, Canada

This protocol describes a retrospective cohort study linking Western Australia (WA) perinatal records (1991–2023) to ERA5 climate data at SA2 resolution, to (1) test multiple heatwave definitions against three neonatal outcomes (preterm birth, low birth weight, congenital anomalies) and select a single definition, then (2) apply that definition to a broad set of maternal, neonatal, and child outcomes using GEE, stratified by climate zone, SES, and trimester.

 Overall, the protocol design is appropriate and robust. I believe the proposed aim is not entirely novel but is comprehensive in a way I have not seen in the literature by accounting for multiple heat indices and examining not only fetal but maternal outcomes. There is a distinct advantage in using Western Australian data given the diversity of climates captured by this geographic region. The design choice of using large, linked cohort with GEE for clustering is sound.

 The following is feedback and recommendations I am suggesting to further strengthen the protocol.

*Exposure/heatwave definitions:*

- My main concern with the definition-selection strategy is that it risks discarding humidity-based indices if they don't "win" against the three neonatal outcomes on strength of association alone. Humidity substantially affects thermoregulatory capacity (reduced evaporative cooling), and is frequently underweighted relative to temperature-only metrics in this literature. I recommend the authors commit to presenting results for at least one humidity-inclusive index (e.g., AT or WBGT) alongside the selected definition in the main analysis, regardless of which definition performs best on the three exploratory outcomes, rather than fully setting aside humidity-sensitive measures.

*Outcomes:*

- * *I have concerns about whether some of the defined outcomes will be captured sufficiently by including a stratified trimester-exposure analysis, especially maternal outcomes.
- I feel that examining child outcomes is out of scope given that the primary focus is on pregnancy; moreover, the child health outcomes while important are less directly impactful on sustaining life compared to the maternal and fetal outcomes. I believe removal of this analysis  should be considered.

I recommend the following to ensure scientific soundness of the protocol:

1. Explicit, pre-specified criteria for selecting the final heatwave definition, with a commitment to retaining and reporting a humidity-inclusive index regardless of which definition is chosen as primary.
2. Clarification of the exposure window (whole-pregnancy vs. trimester-specific) used for each maternal outcome, particularly gestational diabetes and pre-eclampsia.
3. Justification for, or narrowing of, the scope of child health outcomes, with precise ICD-based definitions if this component is retained.
4. A stated approach to multiple-comparisons/false-positive risk given the scale of planned analyses (multiple heat indices × multiple outcomes × trimester and subgroup analyses).
5. A complete covariate list, or a clear rule for how covariates are selected per outcome, given that data availability (BMI, ED records) changes across the 1991–2023 study period.

Is the study design appropriate for the research question?

Partly

Is the rationale for, and objectives of, the study clearly described?

Yes

Are sufficient details of the methods provided to allow replication by others?

Yes

Are the datasets clearly presented in a useable and accessible format?

Not applicable

Reviewer Expertise:

Thermal physiology specializing in female health outcomes, with a focus on pregnancy and post-menopause.

I confirm that I have read this submission and believe that I have an appropriate level of expertise to confirm that it is of an acceptable scientific standard, however I have significant reservations, as outlined above.

# Response

**Published:** 2026-10-06

**Authors:** Erin Kelty

**Affiliations:** The University of Western Australia School of Population and Global Health, Perth, Western Australia, Australia

This protocol describes a retrospective cohort study linking Western Australia (WA) perinatal records (1991–2023) to ERA5 climate data at SA2 resolution, to (1) test multiple heatwave definitions against three neonatal outcomes (preterm birth, low birth weight, congenital anomalies) and select a single definition, then (2) apply that definition to a broad set of maternal, neonatal, and child outcomes using GEE, stratified by climate zone, SES, and trimester.

 Overall, the protocol design is appropriate and robust. I believe the proposed aim is not entirely novel but is comprehensive in a way I have not seen in the literature by accounting for multiple heat indices and examining not only fetal but maternal outcomes. There is a distinct advantage in using Western Australian data given the diversity of climates captured by this geographic region. The design choice of using large, linked cohort with GEE for clustering is sound. The following is feedback and recommendations I am suggesting to further strengthen the protocol.

*Exposure/heatwave definitions:*

- My main concern with the definition-selection strategy is that it risks discarding humidity-based indices if they don't "win" against the three neonatal outcomes on strength of association alone. Humidity substantially affects thermoregulatory capacity (reduced evaporative cooling), and is frequently underweighted relative to temperature-only metrics in this literature. I recommend the authors commit to presenting results for at least one humidity-inclusive index (e.g., AT or WBGT) alongside the selected definition in the main analysis, regardless of which definition performs best on the three exploratory outcomes, rather than fully setting aside humidity-sensitive measures.

*We completely agree with this suggestion and are happy to incorporate it into the protocol paper. We have now indicated that we include up to three definitions of heatwaves, to ensure that humidity and other factors are available in the subsequent analysis.* *Outcomes:*

- * *I have concerns about whether some of the defined outcomes will be captured sufficiently by including a stratified trimester-exposure analysis, especially maternal outcomes.

*I am a little unclear if this concern is associated with the sample size (insufficient power to examine uncommon maternal outcomes stratified by trimester) or the way in which outcomes have been captured and they will be examined by trimester/exposure.* *In terms of sample size, the maternal outcomes specified in our protocol (threatened pregnancy loss, gestational diabetes, pre-eclampsia, the onset of labour, and type of delivery) are relatively common. We are expecting to include approximately 1 million neonates. The proportion exposed to heat during pregnancy, will depend on the heat definition selected and the timing of exposure, but the definition of heatwave during pregnancy should not be such that majority of neonates are exposed (>50%) or so uncommon that exposure if rare (<2%). Ensure a sufficient sample size will have to be examined on a case-by-case basis.* *In terms of outcomes and trimester of exposure, this will also be somewhat dependent on the outcome of interest but will be important in terms of understanding periods of vulnerability. For example, in Western Australia gestational diabetes is tested for at approximately 20 weeks gestation and self-reported at the time of birth. Thus, using first trimester exposure or exposure during the first 20 weeks of gestation would be most appropriate. Similarly, while preeclampsia is not usually diagnosed until late in pregnancy (also self-reported at birth), preliminary research has suggested that the heat exposure in early in pregnancy is associated with a stronger association with preeclampsia (Zheng 2026).   *

- I feel that examining child outcomes is out of scope given that the primary focus is on pregnancy; moreover, the child health outcomes while important are less directly impactful on sustaining life compared to the maternal and fetal outcomes. I believe removal of this analysis should be considered.

*We are actually real excited about the child health outcomes included in this study. We believe that this a novel component of our research and is something that has been largely overlooked in the literature thus far. While these outcomes might be less directly impactful in terms of sustaining life, they can still have a huge impact on an individual. * I recommend the following to ensure scientific soundness of the protocol:

1. Explicit, pre-specified criteria for selecting the final heatwave definition, with a commitment to retaining and reporting a humidity-inclusive index regardless of which definition is chosen as primary.

*We have committed to including at least one humidity related index as a heatwave definition. The criteria specified includes the strength of association with the predefined outcomes, correlation with other measures, portion of exposed pregnancy identified (overall and by climate zone), ease of interpretation and use within the literature and policy. The group that will decide upon the definition includes epidemiologists, biostatisticians, climate scientists, clinicians, and basic scientists. Without having a good understanding of how each measure will perform in our context, it is difficult to be more explicit about the criteria.  *

1. Clarification of the exposure window (whole-pregnancy vs. trimester-specific) used for each maternal outcome, particularly gestational diabetes and pre-eclampsia.

*We have provided more information on the exposure windows for gestational diabetes and preeclampsia. *

1. Justification for, or narrowing of, the scope of child health outcomes, with precise ICD-based definitions if this component is retained.

*We have decided to keep the broad scope of child health outcomes. We have done this as we believe much of the novelty of our study will be in this area. Thus far the research on prenatally exposure to heat has focused on neonatal outcomes, with child health largely overlooked. We have also kept the scope broad to allow flexibility as this area of research grow in the coming years. ICD codes will be developed on a project basis with the help of coding specialists, specialists in the relevant field, and the existing literature. *  

1. A stated approach to multiple-comparisons/false-positive risk given the scale of planned analyses (multiple heat indices × multiple outcomes × trimester and subgroup analyses).

*A statement on multiple-comparisons/false-positives has been added to the end of the analysis section of the methods. *

1. A complete covariate list, or a clear rule for how covariates are selected per outcome, given that data availability (BMI, ED records) changes across the 1991–2023 study period.

*The list of available and potential co-variates is extensive. In addition to maternal characteristics and demographics, using the hospital and ED data we have access to a wide range of maternal diagnoses (e.g. mental health condition, cardiovascular disease), that could be used in the analysis depending on the outcome of interest. Additionally, for child health outcomes, outcomes at birth may be relevant covariates. I have provided more information in the statistical section about the selection of covariates.*

# Reviewer report: Reviewer response for version 1

**DOI:** 10.21956/wellcomeopenres.27580.r144710

**Published:** 2026-02-11

**Authors:** Franca Rusconi

**Affiliations:** 1 Azienda USL Toscana Nord Ovest, Pisa, Italy

The aim of this protocol study is to investigate the impact of exposure to heatwaves during pregnancy on maternal, neonatal, and child health outcomes. To achieve this, babies born in Western Australia between 1991 and 2023 will be identified from perinatal records and linked to weather data. The commonly used definitions of heatwave exposure will be tested against three neonatal outcomes: pre-term birth, low birth weight, and congenital anomalies. A single definition will then be selected to examine maternal, neonatal, and child health outcomes, using linked hospital, emergency department, and educational assessment data, as well as data from the congenital anomalies and death registers.

 Examining the association between exposure to heatwaves in pregnancy and maternal, neonatal, and child outcomes is important given the increasing frequency of heatwaves.

 However, the study protocol has some major weaknesses. While the section on exposure is well written and goes into detail, the introduction lacks important information on previous papers on this topic. The information provided is also confusing (see the main points for the introduction). The description of several outcomes in neonates and in childhood is also confused (see the main points for methods-outcomes). Furthermore, it is highly questionable whether important childhood outcomes such as the development of the central and immune systems can be studied using administrative data.  Consistent with this, the authors provide minimal detail in the Methods section on the definition of the outcomes to be studied, and these are not discussed in the Introduction with reference to the literature.

 Finally, I understand that not all heatwave definitions can be included in the final analysis, but I am a little concerned about selecting a single definition based on associations with three neonatal outcomes: preterm birth, low birth weight, and congenital anomalies. Also, I am not sure what 'based on association' means: association with all three outcomes? Or the strength of the association?

**Main points**

**Introduction**

 Regarding the association between heat exposure and preterm birth and low birth weight, only one paper is cited. However, there are several original papers, as well as meta-analyses, examining this relationship. The authors should cite relevant papers from high-, middle- and low-income countries, including those from Australia. The main results and areas of uncertainty should be reported and discussed. Surprisingly, some of these papers are cited for the first time in the 'Methods' section, both in the text and in Table

 1. In addition, a recent systematic review (Lakhoo DP et al., Nat Med, 2025) is cited in the 'Introduction' section with reference to congenital anomalies. This same review is also cited in the 'Methods' section under 'Outcomes', where it states: 'These outcomes (preterm birth, low birth weight, and congenital anomalies) are the most commonly studied neonatal outcomes, with substantial, robust evidence supporting their association with heatwave exposure'. If the authors believe that Lakhoo et al.'s systematic review and meta-analysis already demonstrate "robust evidence", the aim of this paper is largely confirmatory. This should be discussed when the authors review the relevant papers that are already available (see above). In addition, at the end of the introduction, it should be stated that, with respect to the study of the relationship between heatwaves and the main neonatal outcomes, the present protocol is primarily confirmatory. 

 The protocol aims to assess child health outcomes and hospital and ED presentations, but these are not mentioned in the introduction. The authors should cite and discuss any relevant previous papers on these outcomes, if available, or state that there are no data.

 The authors state that 'in non-pregnant individuals, multiple socioeconomic factors have been identified that either exacerbate or mitigate the association between heat exposure and health outcomes'. These data are also available for heat exposure in pregnancy. The relevant papers should be cited and discussed.

 Environmental factors (e.g. humidity, wind speed and air pollution) are discussed alongside the timing/trimester of exposure. This is confusing. The authors should discuss these topics separately, citing and discussing papers that examine the timing of exposure and neonatal outcomes.

 A literature search should be conducted on the role of environmental factors in pregnancy and their relationship with temperature and neonatal outcomes. Papers should be cited and discussed if available. In addition to the environmental factors listed by the authors, the role of residential greenness in offsetting the negative impact of heat waves during pregnancy on birth outcomes has been investigated in various countries, including Australia.  Even if the authors are not going to study residential greenness themselves, it should at least be mentioned among the environmental factors, with the relevant papers cited. Papers on the interaction between PM, residential greenness and exposure to heat in pregnancy and neonatal outcomes are surprisingly cited in the 'Methods' section (see references 33 and 34).

**Protocol**

 The data sources should be reported separately for the population studied, the exposures and the outcomes.

 For exposure and indices of health, please state which of these have previously been used to study the relationship between heat exposure during pregnancy and the outcomes of the protocol. As mentioned in the comments on the introduction, previous papers on this topic are cited for the first time in Table 1, but no explanation is provided in the text. 

 Outcomes.

 The difference between the three outcomes of interest (pre-term birth, low birth weight (<2,500 g) and congenital anomalies) and the other neonatal outcomes should be made clear. This is only explained in the statistical analysis. In other words, in this chapter, the authors should state more clearly that all the heatwave indices will be tested for these outcomes, while only one heatwave index will be studied for the other neonatal outcomes. Among the other outcomes: I don’t understand the difference between primary and secondary outcomes. For example, if length of gestation is a primary outcome, the percentage of preterm births (before 37 weeks), very preterm births (before 32 weeks) and extremely preterm births (before 28 weeks) cannot be secondary outcomes. The same applies to birth weight. It would be better to refer to these as 'other neonatal outcomes' and specify for 'length of gestation' (<37 weeks; < 32 weeks etc.). The same applies to birth weight: low birth weight and very low birth weight. As for low birth weight for gestational age, the authors should provide a definition.

 Development of the central nervous system: I understand that this outcome is difficult to study using administrative data, but  'mental health admissions, literacy and numeracy scores' are not reliable indicators of central nervous system development. What is the definition of a mental health admission? The authors should specify which problems these are according to the ICD (International Classification of Diseases). Literacy and numeracy scores cannot have the same relevance as mental health problems. Furthermore, how can these outcomes be studied without considering confounding factors such as very low birth weight, prematurity and specific diseases that can affect the development of the nervous system, and are also related to exposure to heat in pregnancy?

 Following the same line of reasoning: the development of the immune system cannot be studied by taking into account hospital and ED presentations for infection(which infections?), allergies (which allergies?), asthma and anaphylaxis (to note: anaphylaxis is a manifestation of allergy).

**Statistical analysis.**

 'Accounting for mothers with multiple neonates': how will this be done? In addition, it is not clear whether multiple births will be excluded from the analysis.

 The analysis will also be performed stratified by environmental factors (e.g. pollution). Are the authors referring to air pollution? I have not seen a definition of how pollution is studied in the chapter “Exposure”.

**Minor points**

**Introduction**

 'While heatwaves are likely to have a negative effect on maternal and neonatal health outcomes in general, the quantitative effect...'. Please replace 'quantitative effect' with 'relationship' or 'strength of association'.

 'The proposed research will examine the relationship between heatwaves, heat stress, and pregnancy outcomes'; please delete 'heat stress' or provide a definition.

**Protocol**

 “Climate data from ERA5 will be mapped to birth records using geocoding at the level of Statistical Area 2 (SA2)”: please explain in more detail what is Statistical Area 2.

Is the study design appropriate for the research question?

Partly

Is the rationale for, and objectives of, the study clearly described?

No

Are sufficient details of the methods provided to allow replication by others?

No

Are the datasets clearly presented in a useable and accessible format?

Not applicable

Reviewer Expertise:

Pediatric epidemiology; environmental factors and neonatal and child health

I confirm that I have read this submission and believe that I have an appropriate level of expertise to state that I do not consider it to be of an acceptable scientific standard, for reasons outlined above.

# Response

**Published:** 2026-07-15

**Authors:** Erin Kelty

**Affiliations:** The University of Western Australia School of Population and Global Health, Perth, Western Australia, Australia

**Thank you for reviewing our manuscript and providing such valuable feedback. Please find below our response to each of the points raised.**

*The aim of this protocol study is to investigate the impact of exposure to heatwaves during pregnancy on maternal, neonatal, and child health outcomes. To achieve this, babies born in Western Australia between 1991 and 2023 will be identified from perinatal records and linked to weather data. The commonly used definitions of heatwave exposure will be tested against three neonatal outcomes: pre-term birth, low birth weight, and congenital anomalies. A single definition will then be selected to examine maternal, neonatal, and child health outcomes, using linked hospital, emergency department, and educational assessment data, as well as data from the congenital anomalies and death registers.* *Examining the association between exposure to heatwaves in pregnancy and maternal, neonatal, and child outcomes is important given the increasing frequency of heatwaves.* *However, the study protocol has some major weaknesses. While the section on exposure is well written and goes into detail, the introduction lacks important information on previous papers on this topic. The information provided is also confusing (see the main points for the introduction). The description of several outcomes in neonates and in childhood is also confused (see the main points for methods-outcomes). Furthermore, it is highly questionable whether important childhood outcomes such as the development of the central and immune systems can be studied using administrative data.  Consistent with this, the authors provide minimal detail in the Methods section on the definition of the outcomes to be studied, and these are not discussed in the Introduction with reference to the literature.* *Finally, I understand that not all heatwave definitions can be included in the final analysis, but I am a little concerned about selecting a single definition based on associations with three neonatal outcomes: preterm birth, low birth weight, and congenital anomalies. Also, I am not sure what 'based on association' means: association with all three outcomes? Or the strength of the association?*

**Main points**

**Introduction**

*Regarding the association between heat exposure and preterm birth and low birth weight, only one paper is cited. However, there are several original papers, as well as meta-analyses, examining this relationship. The authors should cite relevant papers from high-, middle- and low-income countries, including those from Australia. The main results and areas of uncertainty should be reported and discussed. Surprisingly, some of these papers are cited for the first time in the 'Methods' section, both in the text and in Table.*

 We thank the reviewer for this comment. The paper originally cited for pre-term birth, stillbirth, and low birth weight was a systematic review, which synthesised findings from multiple original studies. However, in response to this suggestion, we have expanded the citations to include a more recent systematic review with meta‑analysis (Chersich, Pham et al. 2020), as well as the review by Lakhoo et al. 2025. These reviews examine associations between heat exposure and adverse birth outcomes, including preterm birth and low birth weight, across high‑, middle‑, and low‑income countries, including Australia.

*In addition, a recent systematic review (Lakhoo DP et al., Nat Med, 2025) is cited in the 'Introduction' section with reference to congenital anomalies. This same review is also cited in the 'Methods' section under 'Outcomes', where it states: 'These outcomes (preterm birth, low birth weight, and congenital anomalies) are the most commonly studied neonatal outcomes, with substantial, robust evidence supporting their association with heatwave exposure'. If the authors believe that Lakhoo et al.'s systematic review and meta-analysis already demonstrate "robust evidence", the aim of this paper is largely confirmatory. This should be discussed when the authors review the relevant papers that are already available (see above). In addition, at the end of the introduction, it should be stated that, with respect to the study of the relationship between heatwaves and the main neonatal outcomes, the present protocol is primarily confirmatory.*

 I think there might be a miscommunication, we are not solely interested by pre-term birth, low birth weight, and congenital anomalies, but will be examining a wide range of outcomes with varying levels of pre-existing evidence. Within these three outcomes, there is still sufficient gaps in the evidence to warrant exploring them further e.g. does the association vary between climate zones? do Australia Indigenous persons have a different susceptibility to prenatal heat exposure?

*The protocol aims to assess child health outcomes and hospital and ED presentations, but these are not mentioned in the introduction. The authors should cite and discuss any relevant previous papers on these outcomes, if available, or state that there are no data.*

 Thank you for this helpful suggestion. We have revised the Introduction to include child health outcomes and healthcare utilisation (hospitalisations and emergency department presentations). Specifically, we have added a brief summary of the limited but emerging literature in this area, for example: “Emerging evidence also suggests that prenatal heat exposure may have longer-term implications for child health, including increased risk of respiratory conditions such as asthma and pneumonia, as well as cardiometabolic outcomes including elevated blood pressure (Brink, Lakhoo et al. 2024). However, the evidence base for these outcomes remains comparatively limited.”

*The authors state that 'in non-pregnant individuals, multiple socioeconomic factors have been identified that either exacerbate or mitigate the association between heat exposure and health outcomes'. These data are also available for heat exposure in pregnancy.The relevant papers should be cited and discussed.*

 We thank the reviewer for this comment. We have revised the text to incorporate pregnancy‑specific evidence on socio‑economic disparities in heat exposure. “In both non‑pregnant and pregnant populations, multiple socio‑economic factors have been identified that either exacerbate or mitigate the association between heat exposure and adverse health outcomes. Poverty and low socio‑economic status have been associated with an increased risk of mortality during heatwaves in non‑pregnant populations, likely related to reduced access to air‑conditioning and green spaces, both of which can help to mitigate the effects of heat. Additionally, socio‑economic disparities can shape vulnerability to heat exposure during pregnancy, with associations between heat and preterm birth reported to be strongest among lower socio‑economic groups (Chersich, Pham et al. 2020) and elevated risks observed among socially marginalised populations, including African American mothers in the United States (Syed, O’Sullivan et al. 2022).”

*Environmental factors (e.g. humidity, wind speed and air pollution) are discussed alongside the timing/trimester of exposure. This is confusing. The authors should discuss these topics separately, citing and discussing papers that examine the timing of exposure and neonatal outcomes.*

 We respectfully disagree with this suggestion. The variables noted (environmental conditions, socio‑demographic characteristics, and timing or trimester of exposure) are discussed together intentionally within a single, integrative paragraph that focuses on factors that may modify the association between heat exposure during pregnancy and neonatal outcomes. The aim of this paragraph is to synthesise factors that have been identified in the literature as contributing to variability in observed associations between heat exposure in pregnancy and neonatal outcomes. Additionally, studies examining the timing of exposure in relation to neonatal outcomes are already cited and discussed toward the end of this paragraph, ensuring that this aspect is addressed explicitly within the broader synthesis.

*A literature search should be conducted on the role of environmental factors in pregnancy and their relationship with temperature and neonatal outcomes. Papers should be cited and discussed if available.*

 Thank you for this suggestion. We respectfully note that environmental factors are already incorporated into the Introduction. For example, we state: “Similarly, high humidity dramatically increases the physiological strain that is induced by exposure to high temperature, reducing the efficacy of evaporative cooling, while air pollution (including from wildfires) has also been demonstrated to increase the impact of heatwaves on health outcomes (21).” A more descriptive details of the role of environmental factors in pregnancy is beyond the scope of the introduction and threatens to make the introduction longer than necessary.

*In addition to the environmental factors listed by the authors, the role of residential greenness in offsetting the negative impact of heat waves during pregnancy on birth outcomes has been investigated in various countries, including Australia.  Even if the authors are not going to study residential greenness themselves, it should at least be mentioned among the environmental factors, with the relevant papers cited. Papers on the interaction between PM, residential greenness and exposure to heat in pregnancy and neonatal outcomes are surprisingly cited in the 'Methods' section (see references 33 and 34).*

 Thanks for your comment. Residential greenness is, in fact, briefly acknowledged in the manuscript in relation to socio‑demographic vulnerability to heat. Specifically, we note that reduced access to green spaces may contribute to increased heat‑related risk among socio‑economically disadvantaged populations (“Poverty and low socio‑economic status have been associated with an increased risk of mortality during heatwaves ^17^ , likely related to reduced access to air‑conditioning and green spaces, both of which can help to mitigate the effects of heat ^18^ ^–^ ^20^ .”). In the present study, however, we do not have access to epidemiological data on residential greenness and therefore do not assess this exposure directly. For this reason, we chose not to expand the discussion of residential greenness within the section on environmental factors, as doing so may imply that this variable is formally examined in the current analysis. We aimed to keep the introduction closely aligned with exposures and modifiers that could be directly evaluated within our data. Regarding references 33 and 34, these studies are cited in the Methods section specifically in relation to their findings on elevated nighttime temperatures and their methodological relevance to temperature exposure assessment, rather than for their results concerning residential greenness.

**Protocol**

*The data sources should be reported separately for the population studied, the exposures and the outcomes.*

 We thank the reviewer for this suggestion. We have now included in the Data Source section were the population, exposure and outcomes data is being sourced.  

*For exposure and indices of health, please state which of these have previously been used to study the relationship between heat exposure during pregnancy and the outcomes of the protocol. As mentioned in the comments on the introduction, previous papers on this topic are cited for the first time in Table 1, but no explanation is provided in the text.*

 The references used in Table 1 are examples of where each measure has been used previously in a study examining heat exposure in pregnancy. I have changed the heading to make this clearer. I’m not sure it would be feasible to list all health indices have been studied using each exposure measure, at least not without a systematic review.

**Outcomes** *The difference between the three outcomes of interest (pre-term birth, low birth weight (<2,500 g) and congenital anomalies) and the other neonatal outcomes should be made clear. This is only explained in the statistical analysis. In other words, in this chapter, the authors should state more clearly that all the heatwave indices will be tested for these outcomes, while only one heatwave index will be studied for the other neonatal outcomes. Among the other outcomes: I don’t understand the difference between primary and secondary outcomes. For example, if length of gestation is a primary outcome, the percentage of preterm births (before 37 weeks), very preterm births (before 32 weeks) and extremely preterm births (before 28 weeks) cannot be secondary outcomes. The same applies to birth weight. It would be better to refer to these as 'other neonatal outcomes' and specify for 'length of gestation' (<37 weeks; < 32 weeks etc.). The same applies to birth weight: low birth weight and very low birth weight. As for low birth weight for gestational age, the authors should provide a definition.*

 Thank you for this constructive comment. We agree that the distinction between outcome groupings and their definitions should be clearer. We have clarified this in the outcomes section, ensuring a clear difference between the outcomes used in the initial analysis of heat definitions and the subsequent analysis of outcomes as the main focus of the study. *Development of the central nervous system: I understand that this outcome is difficult to study using administrative data, but 'mental health admissions, literacy and numeracy scores' are not reliable indicators of central nervous system development.*

 Thank you for this important comment. We agree that the term “central nervous system development” was not appropriate in this context and may have been misleading. We have removed this terminology.

*What is the definition of a mental health admission? The authors should specify which problems these are according to the ICD (International Classification of Diseases). Literacy and numeracy scores cannot have the same relevance as mental health problems. Furthermore, how can these outcomes be studied without considering confounding factors such as very low birth weight, prematurity and specific diseases that can affect the development of the nervous system, and are also related to exposure to heat in pregnancy?*

 Very broadly, mental health admission will include admission with an ICD-10 F code and admissions to a psychiatric ward. However, not all F codes will be relevant to our study population (e.g. codes relating to dementia and Alzheimer’s disease are unlikely to be relevant to our young study population or mental health conditions with known causes). Additionally, mental health conditions will be examined by type. The details of this are currently in development with coding experts and will be presented in the relevant paper. With respect to confounding, we agree that factors such as prematurity, very low birth weight, and other conditions affecting early development are critical considerations. These variables will be accounted for within the analytical framework.

*Following the same line of reasoning: the development of the immune system cannot be studied by taking into account hospital and ED presentations for infection (which infections?), allergies (which allergies?), asthma and anaphylaxis (to note: anaphylaxis is a manifestation of allergy).*

 In the revised manuscript, we have refined the language to clarify that these outcomes are immune -related conditions. We have removed anaphylaxis from the list. Immune related conditions are likely to be as per our previously work published in JAMA Open ( https://jamanetwork.com/journals/jamanetworkopen/fullarticle/2814019). The ICD-10 codes used in this component of the research will be published in the relevant manuscript, once it has been finalized.

**Statistical analysis.**

*'Accounting for mothers with multiple neonates': how will this be done? In addition, it is not clear whether multiple births will be excluded from the analysis. The analysis will also be performed stratified by environmental factors (e.g. pollution). Are the authors referring to air pollution? I have not seen a definition of how pollution is studied in the chapter “Exposure”.*

 Where appropriate, generalized estimating equations will be used so that neonates can be clustered by mother in the analysis. This has been included in the statistical section. We have removed reference to air pollution in the statistical analysis section, as we are still working on this data for the study.

**Minor points**

**Introduction**

*'While heatwaves are likely to have a negative effect on maternal and neonatal health outcomes in general, the quantitative effect...'. Please replace 'quantitative effect' with 'relationship' or 'strength of association'.*

 This sentence has already been changed based on an earlier comment.

*'The proposed research will examine the relationship between heatwaves, heat stress, and pregnancy outcomes'; please delete 'heat stress' or provide a definition.*

 This has been removed.

**Protocol**

*“Climate data from ERA5 will be mapped to birth records using geocoding at the level of Statistical Area 2 (SA2)”: please explain in more detail what is Statistical Area 2.*

 We have added a brief definition of SA2.

# Reviewer report: Reviewer response for version 1

**DOI:** 10.21956/wellcomeopenres.27580.r144271

**Published:** 2026-01-21

**Authors:** İrem Özten Dalkıran

**Affiliations:** 1 University of Health Sciences, Istanbul, Turkey

**Journal Name:** Wellcome Open Research

**Title:** The impact of heatwaves on maternal, neonatal and child health outcomes in Western Australian climate zones: A protocol paper for a retrospective cohort study

**Revision Type: **Major Revision

 Thank you for the opportunity to review this important and ambitious protocol. The study addresses a highly relevant public health question and has several notable strengths, including the very large population-based cohort, the use of high-quality linked administrative datasets, and the comprehensive approach to characterising heat exposure using multiple indices.

 The protocol is generally well written and the scientific rationale is strong. However, I have several major and minor comments that I believe should be addressed in order to further improve conceptual clarity, methodological transparency, robustness, and interpretability.

**A. Introduction / Conceptual Framework**

**1. Clear articulation of the literature gap and contribution of the protocol**

 While the protocol is ambitious and methodologically rich, the Introduction does not yet sufficiently and explicitly articulate **the specific gap(s) in the existing literature that this study is designed to address**.

 Based on the current design, the study appears to offer several important contributions, including:

- a systematic, head-to-head comparison of multiple heatwave definitions within the same population and dataset,
- an examination of differential vulnerability across diverse climate zones within a single jurisdiction,
- and an extension of the evidence base beyond birth outcomes to longer-term child health and development.

However, these contributions are not currently framed clearly as responses to concrete gaps in the literature. I strongly recommend that the authors revise the Introduction to more explicitly state:

- what is currently missing or inconsistent in the existing evidence,
- why this creates a problem for interpretation, comparability, or translation into practice,
- and how this specific protocol is designed to address these gaps.

**2. Clinical and public health relevance: why this protocol is needed**

 While the Introduction establishes heat exposure in pregnancy as an important public health issue, it does not yet sufficiently articulate the **clinical and preventive relevance** of the proposed work.

 It would strengthen the manuscript to more explicitly explain:

- what concrete clinical, public health, or policy decisions this study is intended to inform,
- how the results could contribute to risk stratification, early warning systems, clinical guidance, or preventive interventions,
- and how identifying vulnerable subgroups, sensitive time windows, and appropriate heatwave definitions could help **reduce risks and adverse outcomes** in practice.

**3. Broader conceptual framework: primary and secondary pathways of climate change impacts**

 The Introduction currently frames heat exposure largely as a direct physiological stressor. However, climate change affects maternal, pregnancy, and neonatal health through **both primary (direct) and secondary (indirect) pathways**.

 Primary pathways include direct thermal stress, dehydration, and hyperthermia. Secondary pathways include, but are not limited to, air pollution and wildfire smoke exposure, changes in infectious disease patterns, food and water insecurity, psychosocial stress, and disruptions to healthcare access.

 Given the very broad range of outcomes examined in this protocol, the manuscript would be substantially strengthened by briefly situating heat exposure within this **broader climate–health framework**, even if these secondary pathways cannot be directly modelled.

**4. Conceptual and physiological framework: pregnancy-specific thermoregulation**

 Although the protocol provides a very detailed and comprehensive list of maternal, neonatal, and child outcomes (including trimester-specific outcomes), the Introduction currently lacks a sufficiently explicit **physiological framework explaining pregnancy-specific thermoregulation and maternal–fetal heat exchange**.

 Given the very broad spectrum of outcomes examined (e.g., preterm birth, stillbirth, congenital anomalies, labour onset, neonatal anthropometry, neurodevelopmental and immune outcomes), it would substantially strengthen the manuscript if the authors more clearly and explicitly described, in the Introduction, the physiological mechanisms and pathophysiological pathways linking heat exposure to these outcomes.

 In particular, I recommend that the authors add a short, structured subsection in the Introduction that explicitly addresses:

- how thermoregulation changes during pregnancy,
- how heat is transferred between mother, placenta, and fetus,
- why pregnancy constitutes a state of increased vulnerability to heat stress,
- and why specific trimesters may be differentially sensitive to heat exposure.

**5. Structure of the Introduction: outcome-specific conceptual background**

 Although the study ultimately focuses on three key neonatal outcomes (preterm birth, low birth weight, and congenital anomalies) to guide the selection of the heatwave definition, the Introduction currently presents the background in a largely general and undifferentiated manner.

 Given the central role of these three outcomes in the analytic strategy, the manuscript would benefit substantially from a more structured Introduction, with **explicit, outcome-specific subsections**, for example:

- Heat exposure and preterm birth
- Heat exposure and fetal growth / low birth weight
- Heat exposure and congenital anomalies

Within each subsection, the authors could briefly summarise the existing epidemiological evidence and the main proposed biological mechanisms, and clarify why these outcomes are particularly relevant and sensitive to heat exposure at different stages of pregnancy.

**6. Conceptual framework: role of climate-related psychological stress**

 While the protocol provides a very strong and comprehensive framework for examining the physiological impacts of heat exposure, it does not currently consider the potential role of climate-related psychological stress (e.g., eco-anxiety, eco-distress, eco-grief) during pregnancy.

 There is a growing literature suggesting that climate change–related worry and chronic environmental stress may affect maternal mental health, stress physiology, sleep, and health behaviours, which in turn may influence pregnancy and neonatal outcomes.

 Although individual-level measures of climate-related psychological stress may not be available in the linked datasets, I recommend that the authors at least acknowledge this pathway in the conceptual framework and discuss it explicitly in the Introduction and/or Discussion as a potential indirect mechanism and an important limitation.

**7. Clarification regarding “acclimatisation” in the EHF framework**

 The protocol refers to physiological “thermal acclimatisation” within the Excess Heat Factor (EHF) framework. While this is an important concept, it should be clarified that this form of acclimatisation reflects **physiological adaptation to heat exposure** and is conceptually distinct from **psychological or emotional adaptation to climate-related threats**.

 In other words, EHF captures short-term physiological thermal adjustment, but does not address climate-related psychological stress, anxiety, or distress (e.g., eco-anxiety, eco-distress, eco-grief). These psychological pathways may independently or indirectly influence maternal stress physiology, health behaviours, and pregnancy outcomes, and should therefore be acknowledged in the conceptual framework and/or limitations.

**B. Methods / Statistical Analysis**

**8. Strategy for selecting the final heatwave definition**

 The protocol proposes testing a large number of heatwave definitions and then selecting a single definition based on associations with three neonatal outcomes (preterm birth, low birth weight, congenital anomalies). This data-driven approach raises concerns about multiple testing, overfitting, and potential selection (cherry-picking) bias.

- The manuscript should clearly specify **predefined criteria** for selecting the final heatwave definition (e.g., predictive performance, consistency across outcomes, biological plausibility, effect size stability).
- It should also be clarified whether this selection is considered **exploratory** or part of the primary analysis framework.

**9. Multiple comparisons and risk of false positive findings**

 Given the very large number of heat exposure definitions, outcomes, trimester-specific analyses, and stratified subgroup analyses, there is a considerable risk of multiple testing and false positive findings.

- The authors should clarify how they plan to handle this issue (e.g., prioritisation of primary outcomes, hierarchical analysis plan, false discovery rate control, or other strategies).
- At minimum, a clear distinction between **primary analyses** and **exploratory analyses** should be stated.

**10. Confounder selection and causal framework**

 The current list of covariates (maternal age, smoking, parity, Aboriginality) seems incomplete for such a complex exposure–outcome relationship.

 Important potential confounders that should be considered or discussed include:

- socioeconomic status (beyond stratification),
- maternal BMI or obesity,
- long-term temporal trends and seasonality,
- air pollution and other environmental co-exposures.

The manuscript would benefit from a more explicit, theory-driven or literature-based confounder selection strategy (e.g., a DAG-informed approach).

**11. Exposure misclassification due to spatial aggregation**

 Exposure assignment is based on SA2-level geocoding using midpoints, with an additional approach for very large SA2 regions.

- This may introduce **non-differential exposure misclassification**, especially in large rural and remote areas, which could bias effect estimates towards the null.
- This issue should be more explicitly discussed, both in the Methods and in the Discussion / Limitations.

**12. Statistical analysis plan needs more detail**

 While the use of Generalised Estimating Equations (GEE) is appropriate, the statistical analysis section currently lacks important details, such as:

- the assumed distribution and link functions,
- how time trends and seasonality will be handled,
- whether exposure will be treated as binary, categorical, or continuous in some analyses,
- how repeated pregnancies and clustering will be addressed in practice.

**C. Abstract**

**13. Use of abbreviations in the Abstract**

 I recommend that **abbreviations be avoided in the Abstract** (or kept to an absolute minimum), in line with common journal practice and to improve readability for a broad audience. All key terms and indices should be written in full in the Abstract.

**Minor Comments**

1. There appears to be a typographical error in the temperature definitions (e.g., “Minimum temperature (Tmax)” should presumably be “Tmin”).
2. The sections describing the detailed formulas for some indices (especially wet-bulb temperature) are very technical and may be more suitable for supplementary material.
3. In the plain language summary, there is a minor grammatical error:

“how climate change if impacting pregnancy” → should be “is impacting”.

1. Please check spelling consistency (e.g., “caesarian” → “cesarean”).

This is a strong and potentially very influential protocol. However, I believe that **a major revision** is required to clarify key methodological decisions, strengthen the conceptual and physiological framework, better articulate the clinical and translational relevance, and more clearly position the study within the existing literature.

Is the study design appropriate for the research question?

Partly

Is the rationale for, and objectives of, the study clearly described?

Partly

Are sufficient details of the methods provided to allow replication by others?

Partly

Are the datasets clearly presented in a useable and accessible format?

Yes

Reviewer Expertise:

Midwifery, Maternal, Neonatal, and Child Health, Perinatal Health, Public Health

I confirm that I have read this submission and believe that I have an appropriate level of expertise to confirm that it is of an acceptable scientific standard, however I have significant reservations, as outlined above.

## References

[1] : The impact of heatwaves on maternal, neonatal and child health outcomes in Western Australian climate zones: A protocol paper for a retrospective cohort study. *Wellcome Open Research* .2026;11: 10.12688/wellcomeopenres.25022.1 10.12688/wellcomeopenres.25022.1 PMC1368774242920423

# Response

**Published:** 2026-07-15

**Authors:** Erin Kelty

**Affiliations:** The University of Western Australia School of Population and Global Health, Perth, Western Australia, Australia

**Thank you for reviewing our article and your considered feedback. Please find below our response to each of the points raised.**

*Thank you for the opportunity to review this important and ambitious protocol. The study addresses a highly relevant public health question and has several notable strengths, including the very large population-based cohort, the use of high-quality linked administrative datasets, and the comprehensive approach to characterising heat exposure using multiple indices.* *The protocol is generally well written and the scientific rationale is strong. However, I have several major and minor comments that I believe should be addressed in order to further improve conceptual clarity, methodological transparency, robustness, and interpretability.*

***A. Introduction / Conceptual Framework***

***1. Clear articulation of the literature gap and contribution of the protocol*** *While the protocol is ambitious and methodologically rich, the Introduction does not yet sufficiently and explicitly articulate **the specific gap(s) in the existing literature that this study is designed to address**.* *Based on the current design, the study appears to offer several important contributions, including:*

- *a systematic, head-to-head comparison of multiple heatwave definitions within the same population and dataset,*
- *an examination of differential vulnerability across diverse climate zones within a single jurisdiction,*
- *and an extension of the evidence base beyond birth outcomes to longer-term child health and development.*

*However, these contributions are not currently framed clearly as responses to concrete gaps in the literature. I strongly recommend that the authors revise the Introduction to more explicitly state:*

- *what is currently missing or inconsistent in the existing evidence,*
- *why this creates a problem for interpretation, comparability, or translation into practice,*
- *and how this specific protocol is designed to address these gaps.*

Thank you for your feedback. We have worked on the introduction to make sure it clearly frames the gaps in the literature and more explicitly covers the points above.

***2. Clinical and public health relevance: why this protocol is needed***

*While the Introduction establishes heat exposure in pregnancy as an important public health issue, it does not yet sufficiently articulate the **clinical and preventive relevance** of the proposed work.* *It would strengthen the manuscript to more explicitly explain:*

- *what concrete clinical, public health, or policy decisions this study is intended to inform,*
- *how the results could contribute to risk stratification, early warning systems, clinical guidance, or preventive interventions,*
- *and how identifying vulnerable subgroups, sensitive time windows, and appropriate heatwave definitions could help **reduce risks and adverse outcomes** in practice.*

The concern raised is already addressed in the current manuscript. The clinical, public health, and policy relevance of this protocol are explicitly articulated in both the Abstract and the Conclusion. In the Abstract, the study is clearly positioned as providing “valuable information for future planning, including targets for intervention,” which directly reflects its intended contribution to decision-making. In the Conclusion, the manuscript further specifies how the findings will inform public health policies and guidelines, support the development of prediction tools, and enable more targeted strategies to mitigate heat-related risks in pregnancy: “The findings from this study will have implications for public health policies and guidelines that relate to heat stress and pregnancy, nationally and globally. These findings will progress a prediction tool of future health burden for each climate zone. By identifying the potential risks that are associated with exposure to heat during pregnancy, more targeted strategies can be developed to mitigate these risks and protect the health of pregnant people and their children….Ultimately, this research may help to inform the development of public health policies that are aimed at reducing the negative impact of heat exposure on pregnancy outcomes.” These statements explicitly cover risk stratification, preventive interventions, and policy translation. Given that this is a protocol paper, it is not appropriate to elaborate in detail on specific applications or implementation pathways beyond what is already stated, as these depend on the study findings. The current level of detail is consistent with standard reporting expectations for protocols and appropriately avoids overinterpretation. For these reasons, no changes have been made.

***3. Broader conceptual framework: primary and secondary pathways of climate change impacts*** *The Introduction currently frames heat exposure largely as a direct physiological stressor. However, climate change affects maternal, pregnancy, and neonatal health through **both primary (direct) and secondary (indirect) pathways**. Primary pathways include direct thermal stress, dehydration, and hyperthermia. Secondary pathways include, but are not limited to, air pollution and wildfire smoke exposure, changes in infectious disease patterns, food and water insecurity, psychosocial stress, and disruptions to healthcare access. Given the very broad range of outcomes examined in this protocol, the manuscript would be substantially strengthened by briefly situating heat exposure within this **broader climate–health framework**, even if these secondary pathways cannot be directly modelled.*

 The concepts raised by the reviewer are partially addressed in the existing Introduction. The manuscript already acknowledges that the effects of heat exposure vary according to socio-demographic factors and co-exposures such as humidity, wind speed, and air pollution, including wildfire-related pollution. The purpose of this section is to describe factors that modify or interact with heat exposure in the context of the proposed analyses. The study itself is explicitly designed to examine heatwave exposure as the primary environmental exposure, rather than the broader range of indirect pathways through which climate change may influence health (e.g., food systems, infectious diseases, or healthcare access). The Introduction has been updated to briefly acknowledge the broader range of indirect pathways through which climate change may influence maternal and child health, while clarifying that the focus of this study is on heatwave exposure as a direct environmental risk factor.

***4. Conceptual and physiological framework: pregnancy-specific thermoregulation***

*Although the protocol provides a very detailed and comprehensive list of maternal, neonatal, and child outcomes (including trimester-specific outcomes), the Introduction currently lacks a sufficiently explicit **physiological framework explaining pregnancy-specific thermoregulation and maternal–fetal heat exchange**.* *Given the very broad spectrum of outcomes examined (e.g., preterm birth, stillbirth, congenital anomalies, labour onset, neonatal anthropometry, neurodevelopmental and immune outcomes), it would substantially strengthen the manuscript if the authors more clearly and explicitly described, in the Introduction, the physiological mechanisms and pathophysiological pathways linking heat exposure to these outcomes.* *In particular, I recommend that the authors add a short, structured subsection in the Introduction that explicitly addresses:*

- *how thermoregulation changes during pregnancy,*
- *how heat is transferred between mother, placenta, and fetus,*
- *why pregnancy constitutes a state of increased vulnerability to heat stress,*
- *and why specific trimesters may be differentially sensitive to heat exposure.*

The points raised by the reviewer regarding pregnancy-specific thermoregulation and maternal–fetal heat exchange are already addressed in the Introduction to the extent appropriate for a protocol paper, and consistent with the current state of the evidence. First, the manuscript explicitly acknowledges the limited understanding of thermoregulation in pregnancy, stating that: “There is a lack of knowledge on thermoregulation in pregnancy, including how effectively pregnant people adapt in different climate contexts and what specific environmental conditions present the biggest thermoregulatory challenge.” This reflects the current evidence base, which remains incomplete and does not yet support a detailed or structured physiological framework of the type suggested. In the first paragraph of the introduction, the manuscript explicitly states that the fetus is entirely dependent on the mother for heat dissipation and temperature stability, which reflects current understanding that fetal thermoregulation is maternally mediated through placental heat exchange. Again, in this same paragraph, we state why pregnancy constitutes a state of increased vulnerability to heat stress: “pregnancy is a state of vulnerability to heat exposure as it is a dynamic period of physiological and behavioural adaptation”. Second, the Introduction already addresses critical windows of susceptibility across pregnancy, including first-trimester vulnerability during organogenesis and associations with congenital anomalies, and third-trimester exposure and its associations with uterine contractility and preterm birth. These statements explicitly link timing of exposure to outcome-specific biological plausibility, which is the central concept relevant to this study design. The primary aim of the Introduction is to provide sufficient biological plausibility to justify the epidemiological analyses, rather than to present a comprehensive physiological review. Expanding this section into a detailed mechanistic framework would go beyond the scope of the protocol and may misrepresent the strength of current evidence. For these reasons, no substantial restructuring has been undertaken. The minor additions made appropriately acknowledge the concepts raised without overstating mechanisms that are not yet well defined.

***5. Structure of the Introduction: outcome-specific conceptual background***

*Although the study ultimately focuses on three key neonatal outcomes (preterm birth, low birth weight, and congenital anomalies) to guide the selection of the heatwave definition, the Introduction currently presents the background in a largely general and undifferentiated manner. Given the central role of these three outcomes in the analytic strategy, the manuscript would benefit substantially from a more structured Introduction, with **explicit, outcome-specific subsections**, for example:*

- *Heat exposure and preterm birth*
- *Heat exposure and fetal growth / low birth weight*
- *Heat exposure and congenital anomalies*

*Within each subsection, the authors could briefly summarise the existing epidemiological evidence and the main proposed biological mechanisms, and clarify why these outcomes are particularly relevant and sensitive to heat exposure at different stages of pregnancy.*

 Thank you for this thoughtful suggestion. We disagree that restructuring the Introduction into outcome-specific subsections is necessary or appropriate for this protocol. While the reviewer correctly notes that preterm birth, low birth weight, and congenital anomalies are used in the initial stage of the analysis to inform the selection of an optimal heatwave definition, this represents only the first step of a broader research program. The protocol describes a large, multi-phase body of work that will subsequently apply the selected exposure definition to examine a wide range of maternal, neonatal, and child health outcomes. For this reason, structuring the Introduction around these three outcomes would overemphasise their role relative to the overall scope of the study and may give the impression that they are the primary or sole outcomes of interest, which is not the case. Instead, the current Introduction is intentionally framed to reflect the broad and exploratory nature of the research program, providing a general overview of heat exposure in pregnancy and its relevance across multiple outcome domains. In addition, detailed outcome-specific summaries of epidemiological evidence and biological mechanisms are more appropriately presented in manuscripts focused on individual outcomes, rather than in a protocol describing a comprehensive and multi-outcome analytical framework. The current structure is therefore considered more appropriate for accurately representing the aims and scope of the study, and no changes have been made.

***6. Conceptual framework: role of climate-related psychological stress*** *While the protocol provides a very strong and comprehensive framework for examining the physiological impacts of heat exposure, it does not currently consider the potential role of climate-related psychological stress (e.g., eco-anxiety, eco-distress, eco-grief) during pregnancy.*

*There is a growing literature suggesting that climate change–related worry and chronic environmental stress may affect maternal mental health, stress physiology, sleep, and health behaviours, which in turn may influence pregnancy and neonatal outcomes.* *Although individual-level measures of climate-related psychological stress may not be available in the linked datasets, I recommend that the authors at least acknowledge this pathway in the conceptual framework and discuss it explicitly in the Introduction and/or Discussion as a potential indirect mechanism and an important limitation.*

 We acknowledge that the pathway described by the reviewer is part of the broader climate–health context; however, it is not directly relevant to the aims or design of the current study. This protocol focuses on heat exposure as a measurable environmental factor using linked administrative and environmental datasets. Individual-level measures of psychological stress, including climate-related stress, are not available within these data and cannot be incorporated into the analyses. This research is part of a large, funded project, that includes a component examining the more personal experiences of pregnant people who have experienced heat exposure. This protocol paper does not have a discussion, but this limitation can be stated in subsequent publications using the study data, where the results can be discussed in relation to climate-related psychological stress (where appropriate).

***7. Clarification regarding “acclimatisation” in the EHF framework*** *The protocol refers to physiological “thermal acclimatisation” within the Excess Heat Factor (EHF) framework. While this is an important concept, it should be clarified that this form of acclimatisation reflects **physiological adaptation to heat exposure** and is conceptually distinct from **psychological or emotional adaptation to climate-related threats**.* *In other words, EHF captures short-term physiological thermal adjustment, but does not address climate-related psychological stress, anxiety, or distress (e.g., eco-anxiety, eco-distress, eco-grief). These psychological pathways may independently or indirectly influence maternal stress physiology, health behaviours, and pregnancy outcomes, and should therefore be acknowledged in the conceptual framework and/or limitations.*

 We have now specified in the discussion that the EHF includes physiological thermal acclimatisation.

***B. Methods / Statistical Analysis*** ***8. Strategy for selecting the final heatwave definition***

*The protocol proposes testing a large number of heatwave definitions and then selecting a single definition based on associations with three neonatal outcomes (preterm birth, low birth weight, congenital anomalies). This data-driven approach raises concerns about multiple testing, overfitting, and potential selection (cherry-picking) bias.*

- *The manuscript should clearly specify **predefined criteria** for selecting the final heatwave definition (e.g., predictive performance, consistency across outcomes, biological plausibility, effect size stability).*
- *It should also be clarified whether this selection is considered **exploratory** or part of the primary analysis framework.*

We agree and have added some additional information in the methods about what would constitute a good heatwave measure for our project. We have also clarified in the methods that this analysis is exploratory.

***9. Multiple comparisons and risk of false positive findings***

*Given the very large number of heat exposure definitions, outcomes, trimester-specific analyses, and stratified subgroup analyses, there is a considerable risk of multiple testing and false positive findings.*

- *The authors should clarify how they plan to handle this issue (e.g., prioritisation of primary outcomes, hierarchical analysis plan, false discovery rate control, or other strategies).*
- *At minimum, a clear distinction between **primary analyses** and **exploratory analyses** should be stated.*

We acknowledge the reviewer's concern regarding the large number of planned analyses and the associated risk of Type I error arising from multiple comparisons. This is an important consideration in studies using large linked administrative datasets. Several aspects of the proposed work mitigate this risk. The analyses are driven by a priori research questions and a pre-specified analytical framework rather than post hoc exploration of associations. Primary outcomes, exposures and subgroups will be defined in advance, reducing the potential for data-driven findings. Additionally, the study is based on a large population-level cohort, enabling precise estimation of effects. Accordingly, interpretation will focus not solely on statistical significance, but also on effect sizes, confidence intervals, consistency of findings across related analyses, and clinical and policy relevance. We will also distinguish between confirmatory and exploratory analyses. Findings from primary analyses will be prioritised, while results from secondary and exploratory analyses will be interpreted more cautiously. Where appropriate, statistical approaches to address multiplicity, such as false discovery rate control or other suitable adjustments, will be considered. Finally, the proposed analyses represent a coherent programme of work addressing related questions across linked datasets rather than an unrestricted search for significant associations. The richness of the linked data allows robust examination of complex healthcare pathways and outcomes, while the planned analytical approach ensures that the risk of spurious findings is minimised and transparently managed.

***10. Confounder selection and causal framework***

*The current list of covariates (maternal age, smoking, parity, Aboriginality) seems incomplete for such a complex exposure–outcome relationship.* *Important potential confounders that should be considered or discussed include:*

- *socioeconomic status (beyond stratification),*
- *maternal BMI or obesity,*
- *long-term temporal trends and seasonality,*
- *air pollution and other environmental co-exposures.*

*The manuscript would benefit from a more explicit, theory-driven or literature-based confounder selection strategy (e.g., a DAG-informed approach).*

 The list of covariates is listed as examples and are not the complete list.

 Given your comment, we have added more examples to this list, but again it is not complete. The covariates used in each analysis will be largely dependent on the outcomes and the cohort selected. Over the study period (1991-2023), there have been gradual changes in collection of data. For example, data on BMI and alcohol consumption during pregnancy only became available in the last 10 years. Similarly, emergency department data is only available from 2003 onwards, while educational testing started in 2008. Thus, the selection of covariates will be dependent on the outcome, and the available covariates/   Confounder selection will be guided by existing literature and standard practice in environmental epidemiology. Given the breadth of outcomes examined in this protocol, a fully specified causal framework (e.g. DAG) is not presented, as confounding structures may differ depending on the specific outcome under investigation. More detailed, outcome-specific modelling strategies will be developed and described in subsequent manuscripts. In response to this comment, we have expanded the examples to more clearly reflect the broader range of covariates to be considered, such as socioeconomic status and environmental co-exposures. Socioeconomic status will be incorporated both through adjustment and stratification where appropriate.

**11. Exposure misclassification due to spatial aggregation**

 Exposure assignment is based on SA2-level geocoding using midpoints, with an additional approach for very large SA2 regions.

- This may introduce **non-differential exposure misclassification**, especially in large rural and remote areas, which could bias effect estimates towards the null.
- This issue should be more explicitly discussed, both in the Methods and in the Discussion / Limitations.

The authors agree that spatial aggregation of exposure at the SA2 level may result in some degree of non-differential exposure misclassification, particularly in large rural and remote regions. This is something that was discussed with the data custodians, however, they were uncomfortably releasing geocoding of a smaller size (e.g. SA1) due to the potential risk of identifying individuals in the cohort as full date of birth had been provided. We have chosen to use the largest town in areas that are likely to be most affected by this. We have provided some more information on the use of SA2 in the methods. Most very large SA2 contain smaller numbers of residents (2,500-5,000) and thus represent a small number of the total population, thus misclassification is likely to be minimal. Misclassification will be reported as a limitation in any papers publishing results from this study.

**12. Statistical analysis plan needs more detail**

*While the use of Generalised Estimating Equations (GEE) is appropriate, the statistical analysis section currently lacks important details, such as:*

- *the assumed distribution and link functions,*
- *how time trends and seasonality will be handled,*
- *whether exposure will be treated as binary, categorical, or continuous in some analyses,*
- *how repeated pregnancies and clustering will be addressed in practice.*

The assumed distribution and linked function will be largely dependent on the outcome of interest. Where appropriate year of birth and seasonality will be included as covariates. Heat exposure is a binary outcome (now been specified in the statistical analysis section). GEE allow for multiple babies per mother to be factored into the analysis, this has now been explained in the statistical analysis section.

***C. Abstract***

***13. Use of abbreviations in the Abstract***

*I recommend that **abbreviations be avoided in the Abstract** (or kept to an absolute minimum), in line with common journal practice and to improve readability for a broad audience. All key terms and indices should be written in full in the Abstract.*

 The abbreviation for Western Australia has been removed from the abstract and written in full throughout the Abstract.

**Minor Comments**

1. *There appears to be a typographical error in the temperature definitions (e.g., “Minimum temperature (Tmax)” should presumably be “Tmin”).*

Thank you, this has been corrected.

1. *The sections describing the detailed formulas for some indices (especially wet-bulb temperature) are very technical and may be more suitable for supplementary material.*

We believe this is a key component of our proposed research and would like to keep it in the main text.

1. *In the plain language summary, there is a minor grammatical error:*

*“how climate change if impacting pregnancy” → should be “is impacting”.*

 Thank you, this has been corrected.

1. *Please check spelling consistency (e.g., “caesarian” → “cesarean”).*

Thank you. To align with Australian/British English, we have changed this to “caesarean”. *This is a strong and potentially very influential protocol.*
